# Bowel ischemia in a baby with unspecified renovascular hypertension: a case report

**DOI:** 10.1186/1752-1947-5-569

**Published:** 2011-12-09

**Authors:** Omar Oda, Mohammad Zamakhshary, Mohammad Al Namshan, Saud Al Jadaan, Hisham Al Shalaan

**Affiliations:** 1 Division of Pediatric Surgery, Department of Surgery, King Fahad National Guard Hospital, King Abdulaziz Medical City, PO Box 22490, Riyadh 11426, Saudi Arabia; 2 Department of Surgery, King Abdulaziz Medical City, King Saud Bin Abdulaziz University for Health Sciences, Mail Code 1446, PO Box 22490, Riyadh 11426, Saudi Arabia; 3 Department of Radiology, King Abdulaziz Medical City, King Saud Bin Abdulaziz University for Health Sciences, Mail Code 1222, PO Box 22490, Riyadh 11426, Saudi Arabia

## Abstract

### Introduction

Renovascular hypertension due to congenital multiple visceral arterial stenoses in neonates is rare. Management is challenging and has not been standardized. Medical control of blood pressure remains the first-line therapeutic approach. However, unwise control of blood pressure in such cases may lead to disastrous situations.

### Case presentation

We present the case of an 18-day-old Saudi girl with hypertension due to unspecified vascular occlusive disease. The hypertension was managed medically by maintaining blood pressure at 'near normal' levels, and this led to bowel ischemia. Our patient survived the short bowel syndrome and is now two years old. She is on full oral feeding and has reached acceptable growth parameters. Her blood pressure has stabilized at around 110/70 mmHg without anti-hypertensive drugs. She has good organ function and walks despite increased narrowing in stenotic areas and complete obliteration of her left iliac and femoral arteries as seen on follow-up computed tomography angiography.

### Conclusions

We suggest keeping blood pressure at the highest levels permissible in similar clinical situations to prevent a state of bowel hypoperfusion. When alternative treatments for congenital multiple visceral arterial stenoses are not feasible, careful medical therapy and a waiting approach for collaterals to develop may be appropriate.

## Introduction

Renovascular disease is an important cause of hypertension in children, and the incidence is reported to be 3% to 10% [1-5]. Medical management remains the first line of treatment [1-9]. However, 'unwise' control of blood pressure (BP) may lead to disastrous situations. We present the case of a baby with congenital multiple visceral arterial stenoses in which medical therapy contributed to the development of bowel ischemia.

## Case presentation

An 18-day-old full-term Saudi girl who had an unremarkable prenatal and family history (as obtained from the parents) and who was born via normal spontaneous vaginal delivery with a birth weight of 2.5 kg was admitted to a provincial hospital with cardiogenic shock. High BP was diagnosed, and she was treated as a case of cardiomyopathy and was discharged home after 10 days on propranolol and captopril. Without a medical report from the previous hospital, her parents brought her to our pediatric emergency room at the age of 45 days with lethargy and poor oral intake. She looked malnourished and hypoactive. Her weight was 2.4 kg. She had a high systolic BP of 114 to 178 mmHg (normal values for this age and weight are from 70 to 80 mmHg) and a diastolic BP of 57 to 82 mmHg (normal values for this age and weight are from 30 to 40 mmHg).

**Figure 1:**
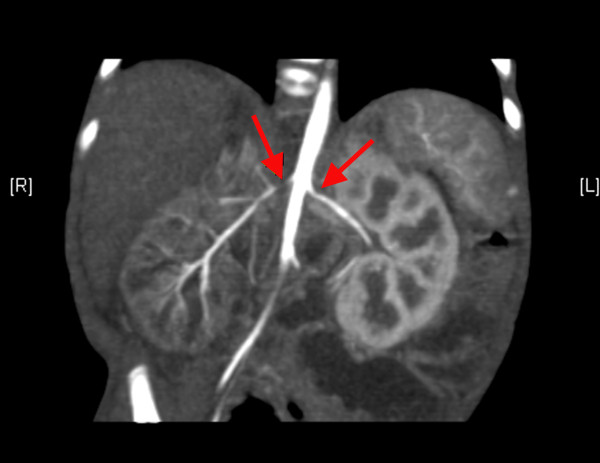
**A computed tomography angiogram shows stenosis of both renal arteries near the ostium (arrows)**.

**Figure 2:**
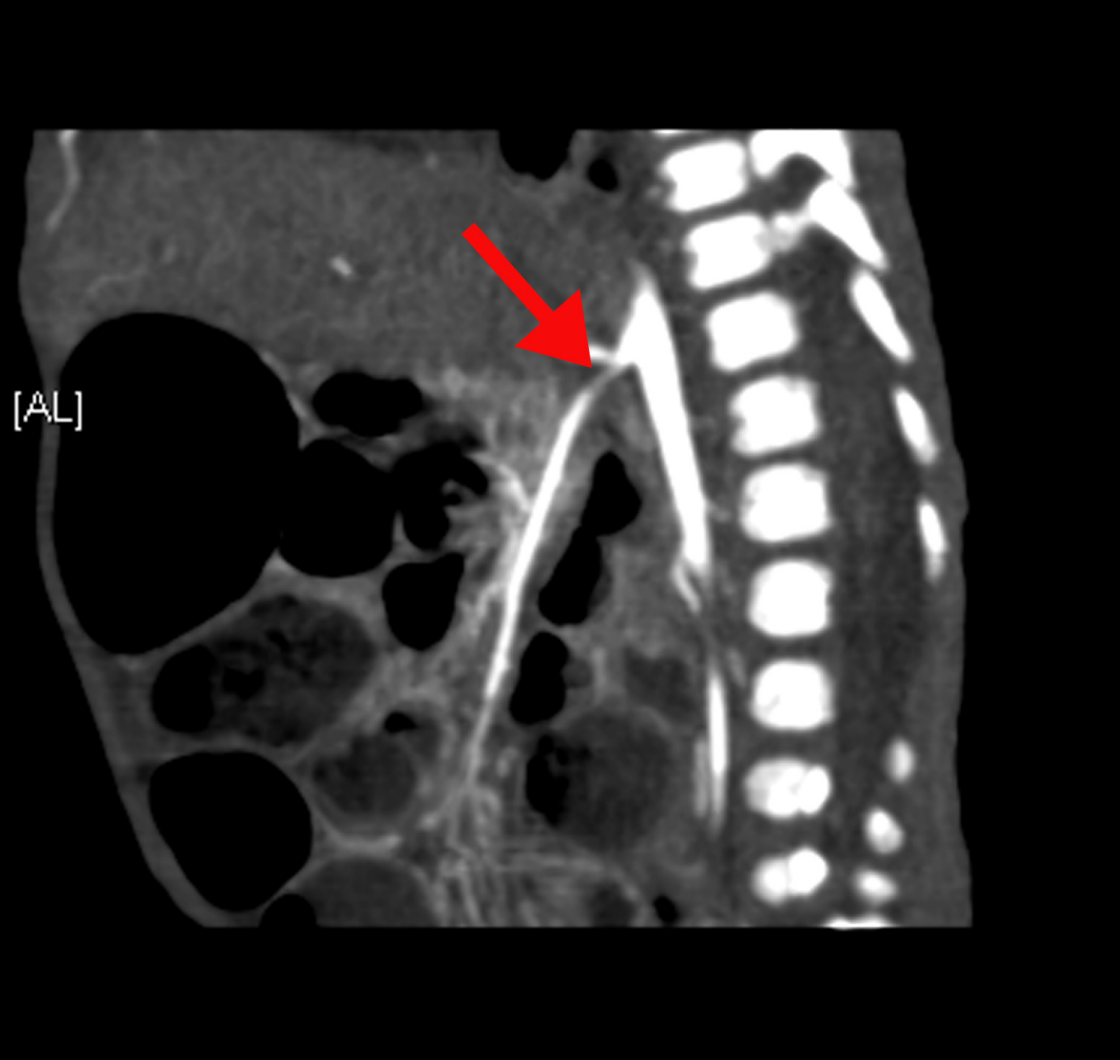
**A computed tomography angiogram shows stenosis of the superior mesenteric artery (arrow)**.

She was admitted to the pediatric intensive care unit and required daily doses of 1.8 mg of hydralazine, 9 mg of propranolol, and 12 mg of captopril to keep her systolic BP in the range of 85 to 142 mmHg and her diastolic BP in the range of 43 to 75 mmHg. Laboratory studies showed the following: a white blood cell count of 32.8 × 10^9 ^cells/L, a hemoglobin level of 102 g/L, a platelet count of 804 × 10^9 ^cells/L, erythrocyte sedimentation rate of 2 mm/hour, normal renal and liver profile results, normal urine analysis results, a serum renin level of 625 nmol/L, a serum cortisol level of 526 nmol/L, and a growth hormone level of 58 μg/L. An echocardiogram showed severe non-obstructive hypertrophy of both ventricles and normal cardiac function. A Doppler ultrasound of her renal arteries revealed severe bilateral renal artery stenosis with a peak systolic velocity of 250 cm/second and a resistive index of 0.89. A computed tomography (CT) angiography revealed multiple arterial stenoses involving both renal arteries near the ostium (Figure 1), the superior mesenteric artery (Figure 2), the celiac artery, the hepatic artery, and both femoral arteries.

Our patient was stabilized on daily doses of 6 mg of hydralazine and 9 mg of propranolol to keep her systolic BP in the range of 97 to 114 mmHg and her diastolic BP in the range of 39 to 54 mmHg. She was discharged home on these medications with a plan to undergo percutaneous transluminal angioplasty (PTA) of the stenosed arteries when she reached a weight of 5 kg. Two weeks after discharge, she presented to our pediatric emergency room with sepsis and greenish vomiting, rectal bleeding, and pneumoperitoneum. A laparotomy revealed bowel necrosis involving her ileum, cecum, and ascending colon. Her necrosed bowel was resected, and a jejunostomy with a mucus fistula at her transverse colon was created. The multidisciplinary team treating her included a pediatric surgeon, a pediatric intensivist, a pediatric nephrologist, a pediatric gastroenterologist, a pediatric geneticist, a pediatric rheumatologist, a pediatric radiologist, and an interventional radiologist. She stayed in hospital for about eight months. The results of a genetic analysis were normal, and metabolic disorders were ruled out. A skin biopsy was normal. The short bowel syndrome was managed successfully. The stoma was closed with a small bowel-to-transverse colon anastomosis. When she reached a weight of 5 kg, two attempts to perform PTA failed because of the very small caliber of her femoral arteries. During this eight-month period, all efforts were directed at keeping her systolic BP between 115 and 150 mmHg to prevent a further episode of bowel hypoperfusion. Later, she was discharged on full oral feeding and 2 mg of hydralazine orally every eight hours and 3 mg of propranolol orally every eight hours as needed if her systolic BP exceeded 150 mmHg.

**Figure 3:**
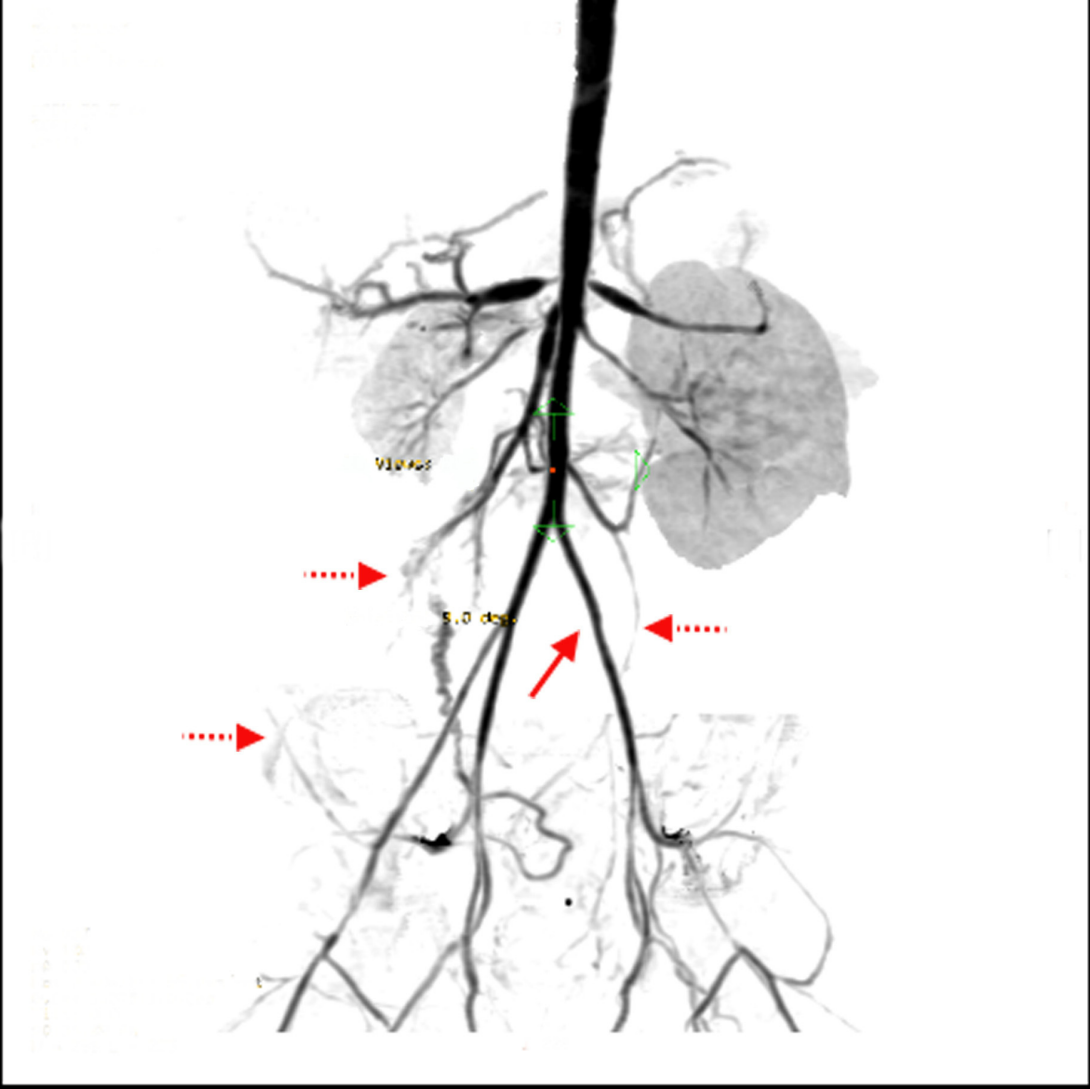
**A follow-up computed tomography angiogram shows complete obliteration of the left external iliac and femoral arteries (arrow) and development of collateral circulation (dotted arrows)**.

At present, she is two years old and has normal cardiac, liver, and renal functions. She is on full oral feeding, and her weight is 11.5 kg. She has not required anti-hypertensive medications for the last six months. A recent CT angiography revealed increased narrowing of both renal arteries, her superior mesenteric artery, her celiac artery, and her hepatic artery and complete obliteration of her left external iliac and left femoral arteries. However, a good set of collateral vessels was seen during the evaluation (Figure 3).

## Discussion

Renovascular hypertension in babies is caused by any one of a large group of vascular disorders. These disorders include renal venous thrombosis, thromboembolism of the renal artery, external compression of the renal artery, fibromuscular dysplasia, neurofibromatosis, Takayasu arteritis, Kawasaki disease, Williams syndrome, mid-aortic syndrome, idiopathic arterial calcification, and a so-called unspecified group of vascular occlusive diseases [1,5]. The management of hypertension is individualized and depends primarily on the causative disorder [2-4]. However, regardless of the causative disease, control of BP is a priority to prevent the possible complications of hypertension. Reproducible BP measurements above 90/60 mmHg are widely accepted as the definition of hypertension in the term neonate [8]. Although many ill neonates are treated for hypotension and hypertension, the normal physiological BP to ensure appropriate organ perfusion is uncertain [3]. Intestinal angina in patients with multiple arterial stenoses is rare. Stanley and colleagues [2] reported only two cases with classic intestinal angina out of 24 cases with splanchnic arterial lesions. Sethna and colleagues [7] reported only one case of bowel ischemia out of 102 cases of idiopathic mid-aortic syndrome. Our patient's condition belonged to the group of unspecified vascular occlusive diseases. Medical control of the BP with a PTA, or vascular reconstructive surgery at a later date, seemed to be an appropriate treatment strategy. The BP was brought to 'near normal' levels. However, in the presence of superior mesenteric artery stenosis, these 'near normal' levels were insufficient to ensure adequate bowel perfusion, and our patient developed bowel ischemia.

When she passed the critical period of sepsis and short bowel syndrome and reached a weight of 5 kg, she underwent two attempts to perform PTA. These attempts were unsuccessful because of the very small diameter of her femoral arteries. Open vascular surgery was unfeasible because of lack of experience. The only choice was to wait and see. Although a radiological follow-up showed increasing narrowing in the stenotic areas, our patient showed clinical improvement over time. This could be explained by the development of collateral circulation seen on follow-up Doppler ultrasound and CT angiography. Sethna and colleagues [7] reported oliguric renal failure in only 4% of 102 cases of idiopathic mid-aortic syndrome and stated that effective collateral circulation develops over time. Srinivasan and colleagues [10] documented the presence of collateral circulation in 51% of the 68 angiograms performed for 43 children with renovascular hypertension due to fibromuscular dysplasia and neurofibromatosis type 1.

## Conclusions

In babies, the management of hypertension caused by unspecified vascular occlusive disease is challenging. A multidisciplinary approach is important. Although intestinal angina is a rare complication, doctors and parents should be aware of it. Parent education is essential to prevent late presentation. Keeping BP at 'high permissible' levels may prevent bowel hypoperfusion. Aggressive angioplastic interventions and open reconstructive surgeries are not indicated when the BP is medically controlled and the organs are functioning normally. Careful medical treatment and waiting for collateral circulation to develop may be appropriate in such difficult clinical situations.

### Consent

Written informed consent was obtained from the patient's next-of-kin for publication of this case report and any accompanying images. A copy of the written consent is available for review by the Editor-in-Chief of this journal.

## Competing interests

The authors declare that they have no competing interests.

## Authors' contributions

HAS interpreted our patient's radiological data. MZ, MAN, and SAJ were major contributors to writing the manuscript. All authors read and approved the final manuscript.
